# Psychological Availability between Self-Initiated Expatriates and Host Country Nationals during Their Adjustment: The Moderating Role of Supportive Supervisor Relations

**DOI:** 10.3389/fpsyg.2017.02049

**Published:** 2017-11-24

**Authors:** Milad Jannesari, Zhongming Wang, Jacob McCall, Boyang Zheng

**Affiliations:** ^1^School of Management, Zhejiang University, Hangzhou, China; ^2^Rutgers-Newark Master of Fine Arts in Creative Writing, Rutgers University–Newark, Newark, NJ, United States; ^3^Department of Psychology and Behavioral Science, Zhejiang University, Hangzhou, China

**Keywords:** adjustment, psychological availability, self-initiated expatriates, host country national, proactive personality, supportive supervisor relations

## Abstract

This research examined the role of psychological availability as a means of psychological engagement between self-initiated expatriates (SIEs) and their host-country nationals (HCNs) colleagues during their work and interaction adjustment. To reveal this process, this study presented the concept of psychological availability, which refers to an individual’s belief that they are physically, cognitively, and emotionally ready or confident to engage the self with their colleagues, as a mediator between proactive personality and adjustment. Also, it investigated the relationship between proactive personality and psychological availability and how it was moderated by supportive supervisor relations. We hypothesized, this relationship would be weakened/strengthened when SIEs and HCNs received low/high level of support from their supervisor. This study was conducted as a quantitative study, data was used from 342 SIEs and 342 HCNs working in mainland China. Our finding supported the hypothesis that psychological availability mediated the relationship between proactive personality and their adjustment to an international work environment; in addition, the relationship between proactive personality and psychological availability would be stronger when the level of superiors relations support is high between SIEs and HCNs. This study demonstrated the value of proactive personality as an antecedent effect and supportive supervisor relations as a moderating effect, and investigated how these factors can lead to a sense of psychological availability and boost psychological engagement between SIEs and HCNs in order to improve the adjustment between them.

## Introduction

Due to the increasing globalization of work environments and enhanced competition between global organizations, the spread of expatriates’ assignments to enable global growth is increasing among global organization (Brookfield Global Relocation Services, 2016). Thus, one of the areas that has caught the attention of international human resource managers is the adjustment of their expatriates to their new situation (Zhang and Dodgson, 2007; Fee et al., 2013; Ren et al., 2014). Specifically, they are coming to realize how they should develop relationships during the adjustment between their expatriates and their local personnel to maintain their competitive advantages (Chen et al., 2002; Toh and DeNisi, 2005; Luo, 2016). This becomes more challenging for them when theses personnel are self-initiated expatriates (SIEs) who are not transferred overseas from a parent company, because they are not pre-trained or prepared to adjust to their new cultural environment and local colleagues (Peltokorpi and Froese, 2013). Although, this is challenging for them to adapt their SIEs employees to the new situation, maintaining an expatriate is costly and complicated process, and if they fail in their tasks this would be even more costly (Toh and DeNisi, 2005). Therefore, global organizations and scholars (Al Ariss and Ozbilgin, 2010; Doherty et al., 2011; Vaiman et al., 2015) have been focusing on SIEs who are rising in number. A SIE is defined as a person who independently chooses to expatriate (Suutari and Brewster, 2000). Their expatriation experiences are riskier than corporate expatriates who are sponsored by organizations to take an international assignment for a specific time and prepared for better interaction with their HCNs colleagues who are from different cultural backgrounds (Peltokorpi and Froese, 2009). Another reason to focus on SIEs is that they are more vulnerable during their adjustment with their local colleagues than traditional organizational expatriates who are selected and trained by headquarters to be ready for this adjustment to their new cultural work situation (Doherty and Dickmann, 2013).

Also, in this research, we direct our attention toward psychological availability. This is a concept that deals with the “sense of having the physical, cognitive, emotional, or psychological resources to personally engage at a particular moment” (Kahn, 1990, p. 714). And psychological availability is considered as one of the psychological conditions that can help individuals to determine how to engage with their roles or colleagues (May et al., 2004). In another word, they defined psychological availability as “one’s ability and motivation to direct psychological resources at the partner” (Danner-Vlaardingerbroek et al., 2013, p. 54). Therefore, this is very important for SIEs and their HCNs colleagues to understand how to be psychologically available for their colleagues and how to have the mental capacity to give more attention to their colleagues while they are adjusting to them (Danner-Vlaardingerbroek et al., 2013). This issue has never been considered before between SIEs and their local colleagues. Especially, at the present time, the researchers mainly focused on how to develop adjustments among SIEs by different career competencies (e.g., Cao et al., 2013), and they have given less attention on how SIEs are psychologically present during particular moments of adjustment. Most SIEs research focuses on unidirectional adjustment from SIEs to HCNs (Nolan and Morley, 2014; Selmer and Lauring, 2015), but this study goes beyond this tradition by considering the process of adjustment from the perspective of both parties (i.e., SIEs to HCNs at the same as from HCNs to SIEs). Traditional expatriate research, which is SIEs-centric, often neglected the roles of HCN colleagues (e.g., Selmer and Lauring, 2014a,b). This study filled this research gap by incorporating HCNs’ perspectives in the process of adjustment. Therefore, based on these arguments we’ve developed our research question: *How do SIEs and HCNs develop their psychological availability as an instrument that can allow them to better adjust to each other?*

The purpose of this study, is to help SIEs and HCNs develop psychological availability as a tool to bolster psychological engagement which can provides guidance on how to adjust with each other. To address this implication this study traced and developed a research model by first and second exploring proactive personality as a potential personality trait that might shape cross-cultural adjustment and psychological availability, respectively, between SIEs and HCNs. According to previous investigation (Crockett, 1962.), personality traits are critical factors in determining how individuals feel, think and how they behave with regards to their occupational mobility, and this study considered the effect of proactive personality on adjustment and psychological availability between two actors. Due to this issue, in the 21st century, careers have become increasingly boundaryless (e.g., Arthur and Rousseau, 1996; Hall, 1996; Sullivan and Arthur, 2006), and individuals are more likely to work with multinational colleagues (Firth et al., 2014) and this is a very important strategy for individuals to behave proactively to have successful psychological engagements with their colleague from a different culture (Sonnentag et al., 2012). Particularly, for SIEs, who unlike those sent by their organization, should be proactive because they need to handle all the difficulties by themselves during their adjustment with their local colleagues (Andresen et al., 2015). Third, we investigated the psychological availability concept, which is considered as “being interpersonally present for the partner and having the mental capacity to actively direct attention to the partner” (Danner-Vlaardingerbroek et al., 2013, p. 53), as a mediator in the relationship between proactive personality and adjustment. Final, we examined the relationship between the concepts of proactive personality and psychological availability would be weakened/strengthened when SIEs and HCNs received low/high level of support from their supervisor. Previous literature also revealed support supervisor relations can predict the quality of the relationship between employees and their supervisor, i.e., high level of support supervisor relations represent mutual understanding, common vision, and respects between them (Kraimer et al., 2001). Also, in cross-cultural adjustment, this concept is one of the critical sources that can increase the ability of SIEs to adjust with new situations or colleagues (Kraimer and Wayne, 2004; Lee et al., 2013).

In sum, we identified the direct and indirect potential antecedents of SIEs and HCNs that contributed to their positive psychological availability to each other, which is an instrument in developing and helping adjustment between them.

### Proactive Personality and Adjustment

Bateman and Crant (1993) defined proactive personality as a concept which refers to an individual’s dispositional ability to take reaction initiativly to their environment changes, Crant (2000) manifested a person with high level of proactive personality, who is initiative, can recognize opportunities and immediate perform until creating positive changes in their environments. Also, there are numbers of cross-cultural studies examining the expatriates during their assignments who behave proactively. Normally, behaving proactively, is considered a positive factor to their adjustments and allows them to easily overcome cultural barriers (Ren et al., 2014). Although, in their studies, the relationship between proactive personality and adjustment has never been generalized to any types of dyads such as supervisor-expatriates or SIEs-HCNs, instead, they emphasized the interaction consists of two sources or actors. Specially, for SIEs-HCNs coming from different cultural backgrounds this may provide them with a big obstacle to managing uncertainty and anxiety among them which has negative effects on them during their work and interaction adjustment (Gudykunst, 1998; Gudykunst and Nishida, 2001).

This study defined the relationship between SIEs and HCNs who act proactively to each other, they may look at cultural barriers between them as disguised opportunities. They don’t look at them as a problem; they take the initiative to find a way to address the problem. Instead of viewing these barriers as roadblocks, these obstacles become their personal challenges to overcome, proactively they act to control their environment in order to have better adjustment between. As well as, in Selmer and Lauring’s (2013) study, showed that those SIEs with a more proactive personality as a competent people who are capable in their decisions and behaviors, likely they are more willing to be socially integrated and develop their relationship with HCNs. Also, Shaffer et al. (2006) declared that when individuals go to a new country where they are unfamiliar with the norms of behavior, their behavior will be more shaped by personal resources in order to determine their adjustment to this new cultural environment. Also, in other practical expatriate studies, it demonstrated that proactive behaviors could reduce the anxiety and uncertainty as cultural barriers and provide socialization among them (Fang et al., 2011). That helps them to feel comfortable when they are interacting and working with each other (Hsu, 2012). In other words, when two actors proactively engage in networking, they are more willing to have frequent interactions with each other (Fang et al., 2011). Therefore, based on above arguments, this study believed and the hypothesized that the proactive personality will be positively related to the adjustment between SIEs and HCNs.

*Hypothesis 1a:* The SIEs with high proactive personality will be associated with better adjustment to their HCN colleagues.
*Hypothesis 1b:* The HCNs with high proactive personality will be associated with better adjustment to their SIE colleagues.

### Proactive Personality and Psychological Availability

Kahn (1990, p. 700) defined psychological availability as the “simultaneous employment and expression of a person’s ‘preferred self’ in task behaviors that promote connections to work and to others, personal presence (physical, cognitive, and emotional) and active, full performances.” In another word, availability of individuals is considered as being psychologically present to others in such a way that confers that person their full focus and attention when they are connecting and interacting with their partner, colleagues, supervisor and etc. (Kahn, 1992). Therefore, Kahn (1992) viewed psychological availability as a behavioral engagement of personal physical, cognitive, and emotional energy into connecting with others. Furthermore, an individual present psychological availability when they are willing or ready to physically engage, cognitively focused, and emotionally connect to others (Danner-Vlaardingerbroek et al., 2013). In another point of view, Ashforth and Humphrey (1995) assimilated the psychological availability as an individual’s engaging the “hands, head, and heart” (p, 110) with others.

Also, in the practical study by Rich et al. (2010), they employed psychological availability as a concept in order to represent individuals’ readiness to personally engage at a certain time. Indeed, they realized that one of the key factors that can enhance an individual’s availability or readiness is that an individual have a high level of confidence in their own capabilities that give more invest of self in the role of personal engagement with their colleagues.

Therefore, this study postulated that the proactive personality would positively influence psychological availability between individuals and their partners, such an individual with the high proactive personality will be more capable of identifying and preventing potential problems that can help to control their psychological personal resources (cognitive, emotional, and physical) that they have to put in when they are working together. It further suggested that the proactive personality is a type of confidence (Grant and Ashford, 2008), which helps SIEs and HCNs to approach and deal with their cultural difference and shapes better behavior toward each other, in such a way with more confidence they feel about their capabilities and status, and they are more willing to feel available and ready to engage fully with each other. Also, Kahn (1990) discussed that controlling and enhancing psychological availability between colleagues correlates to that individual feeling of security during their work with their colleagues, also, being less psychological present for the colleagues display insecurity felt by that individual. As well as, in practical research by Binyamin and Carmeli (2010), it has displayed that stress and anxieties negatively affect psychological availability of employees which in turn reduce their creativities.

Kahn (1990, p. 716) mentioned that “being available was partly a matter of security in abilities and status and maintaining a focus on tasks rather than anxieties.” Due to this inherent aspect of proactive personalities, this facilitates individuals to reduce the influence of stressors during their work task and this can assist them to more focus on relationship with their colleagues or supervisor (Hsu, 2012). Especially, working in a cross-cultural environment is highly stressful and uncertain, which is faced by both SIEs and their local colleagues when they are working together. Therefore, in this environment it’s not sufficient for SIEs and their local colleagues to simply react to each other, indeed, they have to act proactively upon to each other in order to raise the capabilities that they possess which can help to reduce uncertainty between them (Aragon-Correa, 1998; Griffin et al., 2007); they will tend to have more psychological resources to put toward each other’s. In turn, they will likely put more energy to focus attention at their SIE/HCN colleagues when they are communicating with each other. This makes them more physically, emotionally, and cognitively available for each other. Therefore, this study predicted that proactive personality will be positively related to psychological availability between SIEs and HCNs, and we hypothesize thus:

*Hypothesis 2a:* The SIEs with a more proactive personality to be related to higher levels of psychological availability for their HCN colleagues.
*Hypothesis 2b:* The HCNs with more proactive personality and this relates to higher levels of psychological availability for their SIE colleagues.

### Mediating Effect of Psychological Availability

According to this point, we have claimed that proactive personality promotes individuals’ ability to simultaneously engage physically, cognitively, and emotionally in the relationship with others. Also, for this study we developed a model in which psychological availability mediates the relationship between proactive personality and adjustment. Although, in expatriates studies we have found that proactive personality (Ren et al., 2014) are significantly related to adjustment, this study suggested that psychological availability plays a critical role in this relationship in order to promote adjustment between SIEs and HCNs. Also, in practical research, it assumed that to enhance positive behaviors during the interactions between two actors, it requires both to take the perspectives and self-interested behavior toward each other in order to take to assess the quality of the relationship (Rusbult et al., 1991). This quality of the relationship requires psychological resources that are fully or sensitively focused on each other (Yovetich and Rusbult, 1994; Finkel and Campbell, 2001). Also, recent practical study displayed, that psychological availability has a high influence on positive martial behaviors (Danner-Vlaardingerbroek et al., 2013).

In cross-cultural studies, intercultural adjustment has been considered as one of the most critical factors between expatriates and local peoples (Davies et al., 2015). This concept can reveal that to what extent expatriates and local peoples would like to incorporate in work and daily life or how much willing they would interact with each other (Davies et al., 2015; Salamin and Davoine, 2015). For instance, Sonnentag et al. (2012) argued that expatriates who are simultaneously engaged (physically effort to pursue the relationship, being cognitively attentive, and emotionally connected to their local colleagues), are better able to adjust to their host colleagues. Particularly, when SIEs and HCNs behave proactively this might help them to have more readiness or confidence to engage themselves physically, cognitively, and emotionally in their adjustment to each other, and this should provide them with security which will make them more willing to step outside their comfort zone and take more risks in order to more fully engage with each other in their adjustment (Bakker et al., 2005; Molinsky, 2007, 2012). Therefore, this study expected that with proactive personality they can enhance their available psychological resources and create and focus their attention on the other group, finally result in better adjustment for both SIEs and HCNs.

In further discussions, Sonnentag et al. (2012) defined individuals’ psychological availability as being psychologically present in the sense that they are fully linked and attentive to their partners. Also, Kahn (1990) discussed that high level of psychological availability among employees helps to inspire and sustain collaborative behavior and openness, not only within the pair but also between other colleagues, and this can bring their fully open themselves to interactions with each other. In this way, psychologically available SIEs and HCNs will tend to be more willing to learn about each other (i.e., about cultural norms and language) which can help them to know how to function with proper behaviors toward each other which in turn leads to better adjustments between them. Due to above arguments, this study expected that proactive personality may facilitate work and interaction adjustment between SIEs and their local colleagues through being psychological available of the self as reflected by engagement physically, cognitively, and emotionally. And we hypothesized as below:

*Hypothesis 3a:* The SIEs’ psychological availability will mediate the relationships between their proactive personality and adjustment with their HCN colleagues.
*Hypothesis 3b:* The HCNs’ psychological availability will mediate the relationships between their proactive personality and adjustment with their SIE colleagues.

### Moderating Effect of Support Supervisor Relations

Working in the cross-cultural environment has been viewed as a problematic situation for both foreigners and local employees (Van Vianen et al., 2004), because the foreigners in this environment always encounter cultural confusion, strangeness, and language barriers with local employees (Louis, 1980; Tu and Sullivan, 1994). Also, local employees during their work with foreign colleague may experience a fear of the foreign colleagues’ potential to hold higher position in the organization (Jannesari et al., 2016), because of that issues, the foreigners and their local colleagues may not be able to cope with each other. This inability may lead to cope with their colleague on the opposite side of the dyad may lead both of them to experience an intense degree of stress which may lead them to feel distrust, humiliation, failure, anxiety or hostility for their other colleague in the dyad (Mendenhall and Oddou, 1985; Adler, 1991; Coyle and Shortland, 1992; Hippler, 2000). Thus, in order to reduce this failure, Kim et al. (2009) described supervisor support as one of the key element that can help to increase emotional well-being between two individuals from a different culture which can help prevent any psychological distress. Therefore, this study investigated supportive supervisor relationships in this phase of leader-member exchange (LMX) (Liden et al., 1997; Kraimer et al., 2001). Leader-member exchange in cross-cultural studies is regarded as the quality of the interpersonal exchange relationship between foreigners and their supervisors (Kraimer et al., 2001).

Furthermore, expatriates with high-quality LMX relationship may receive information and assistance from their supervisor that will contribute to their adjustment (Kraimer and Wayne, 2004). Also, practical research has manifested the LMX as an element of a social support mechanism that can assist and share information about cultural values and norms, and individuals can soon understand about deviation from cultural norms and fix it (Sorensen, 2002). Thus, by LMX which emphasize on relationship development, individuals can feel more confident from their psychological availability among others and in turns reciprocate correct behavior. Therefore, this study extended this consideration and examined this relationship between local employees and their supervisor while they are working with their foreigner colleagues. Thus, this study predicted supportive supervisor relationship will provide a positive working atmosphere among their foreign and local employees, and it will have an effect on the relationship between SIE’s- HCN’s proactive personality and psychological availability. Therefore, we hypothesize this relationship as below:

*Hypothesis 4:* Relationship between proactive personality and psychological availability would be weakened/strengthened when SIEs and HCNs received low/high level of support from their supervisor.

**FIGURE 1:**
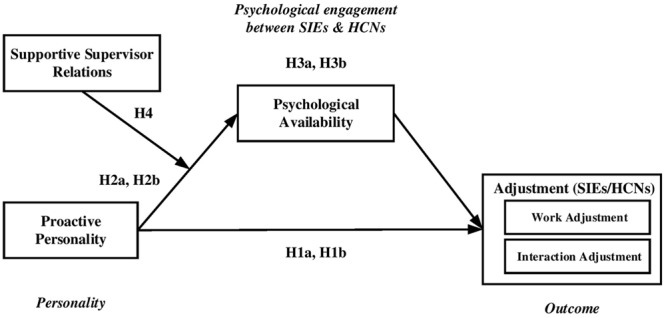
Conceptual model and hypotheses.

**Figure 1** demonstrates the conceptual model for this study.

## Materials and Methods

### Sample and Procedures

This study used a cross-sectional investigation strategy and employed data from SIEs-HCNs dyadic sample. We collected data from SIEs are originally from different nations/regions who are trying to live and work in China mainland. We obtained our samples by identifying multinational companies from two sources China-based MNCs and information from LinkedIn. For this study we sent an email to 820 Multinational companies’ human resource department to ask whether or not they were willing to participate in this study. Those multinational companies that agreed to participate in this study, forwarded our requirement to their SIE employees. After that, we sent them an email that explained our research goals. If they confirmed their status as SIEs based on our questionnaire, and agreed to participate in our study (Selmer and Lauring, 2011), we linked them to the web survey. Also, at the end of the survey, we requested that they give us the name and email of a local colleague with whom she/he would be working and interacting. In the final step, we sent our invitation to HCN colleagues by email, after they agreed we also connected them to online survey system. More than five hundred companies participated in this study. We sent survey invitations to a total of 670 SIEs-HCNs dyadic, we received 342 SIEs-HCNs dyadic valid surveys.

The demographic profile of SIEs: The average age was 41 years (*SD* = 9.65), and were 74% male. 68% held a bachelor degree. For the length of SIEs expatriation, 32% were less than 4 years, 53% had been expatriated between 4 and 8 year, 13% had been expatriated for more than 8 years, and 2% were missing the information. They were from 32 countries and regions: Australia, Austria, Belgium, Brazil, Canada, Croatia, Denmark, England, France, Germany, Hong Kong, Iran, India, Indonesia, Italy, Korea, Malaysia, Mexico, Netherlands, New Zealand, Norway, Switzerland, Scotland, Singapore, South Africa, Sri Lanka, Sweden, Taiwan, Turkey, United Kingdom, United States, and Vietnam. The dyadic tenure or length of the relationship in years between SLEs-HCNs: 11% below 1 year, 56% between 1 and 4 years, 31% more than 4 years, and 2% were missing. The level of position between SIEs-HCNS: 81% of SIEs-HCNs had a peer relationship, 12% of HCNs were supervisors of the SIEs, and 7% of SIEs were supervisors of the HCNs.

The demographic profile of HCNs: The average age was 37 years (*SD* = 7.23), and 62% female, 86% held a bachelor degree. All the HCNs participants in this study were Chinese because we conducted this research in China.

### Measures

#### Proactive Personality

We used 10 items developed by Seibert et al. (1999), which was adopted from Bateman and Crant’s (1993) 17-item scale. For further consideration, a sample item is “I am always looking for better ways to do things with my foreigner/local colleagues.” Items were on a seven-point Likert-type scale ranging from 1 = strongly disagree to 7 = strongly agree. According to Seibert et al. (1999), the Cronbach’s alpha for proactive personality was 0.82.

#### Supportive Supervisor Relations

It was measured by 12 items which we borrowed from Liden and Maslyn’s (1998). A sample item is “I have a supervisor who always defends and supports me if I’m hurt by other colleagues.” All the scales were measured on a 7-point Likert scale ranging from 1 = strongly disagree to 7 = strongly agree. Cronbach’s alpha was 0.93.

#### Psychological Availability

It was measured by using five items employed from May et al. (2004) which were created based on Kahn’s (1990) study. These items were considered if the individuals were physically, cognitively, and emotionally available for adjustment with their partners. A sample item was “I am confident in my ability to deal with problems that come up at work with my foreigner/local colleagues.” All the scales were measured on a 7-point Likert scale ranging from 1 = strongly disagree to 7 = strongly agree. According to May et al. (2004), the Cronbach’s alpha for psychological availability was 0.85.

#### Adjustment

According to Lee (2010) and Lee et al.’s (2013) studies, work and interaction adjustment are the most important two sub-dimensions to predict cross-cultural adjustment. Therefore, we borrowed two sub-dimensions from their study, and borrowed five items from Black and Stephens (1989). The items we employed from Black and Stephens included three items for work adjustment, a sample item is “I am a flexible person who can adjust to working with Local/foreigner colleagues”; and two items for interaction adjustment, a sample item is “I’ve adjusted to interacting with the Local/foreigner colleagues in general.” All the scales were measured on a 7-point Likert scale ranging from 1 = strongly disagree to 7 = strongly agree. Black and Stephens (1989) reported the reliability for interaction adjustment was α = 0.99, and for work adjustment was α = 0.88.

#### Control Variables

This study followed the same model as Varma et al. (2011) and believed that the similarities and differences between two sources or parties have a high influence on their psychological engagement. Hence, we controlled the level of differences and similarities of position in the organizational hierarchy between SIEs and HCNs. Therefore, we conducted two dummy variables: dyadic relationship (1 = dyadic; 0 = SIEs or HCNs as supervisor) and HCN as a supervisor (1 = HCN as supervisor; 0 = peer or SIEs as supervisor). Also, this study controlled for the length of the relationship between SIEs and HCNs because those with longer relationships have better relationship and psychological engagement (Harrison et al., 2004; Muthusamy and White, 2005). Therefore, we measured the dyadic tenure or the length of relationship in years between SIEs and HCNs. In addition, we also examined SIE’s tenure of expatriation, the more years or longer time they’ve work overseas, the more flexible to psychological adjustment they are (Doherty et al., 2013), and this control variable was measured in terms of years that SIEs worked in foreigner countries.

To avoid common method variance (Podsakoff et al., 2003), this study collected data for adjustment concept from leaders or supervisors working with our SIEs and HCNs participants. Thus, we borrowed “Harman’s single factor” techniques in order to control for variance, and the result only presented a 41% of the variance which is not more than 50% of the total variance, therefore, we can claim that there is no issue from common method variance for this study.

### Data Analysis

Before testing hypotheses, this study ran normality test to check all the variables were normally distributed (Kline, 2005). Using Kolmogorov–Smirnov approach, the statistics of proactive personality, supportive supervisor relations, psychological availability, adjustment from SIE’s samples were 0.074 (*p* < 0.05), 0.118 (*p* < 0.01), 0.094 (*p* < 0.01) and 0.091 (*p* < 0.01), respectively; from HCN’s samples were 0.123 (*p* < 0.00), 0.105 (*p* < 0.01), 0.117 (*p* < 0.00) and 0.109 (*p* < 0.00), respectively, indicating the normality of all the variables. The results of Shapiro–Wilk also supported this, given that the statistics of proactive personality, supportive supervisor relations, psychological availability, adjustment from SIE’s samples were 0.979 (*p* < 0.05), 0.940 (*p* < 0.01), 0.966 (*p* < 0.01) and 0.981 (*p* < 0.05), respectively; from HCN’s samples were 0.970 (*p* < 0.00), 0.955 (*p* < 0.00), 0.973 (*p* < 0.01) and 0.974 (*p* < 0.01), respectively.

Then, this study tested hypotheses in two interlinked steps: first, we examined a simple mediation model (hypothesis 1a–3b); second, we examined the proposed moderator variable into the model (hypothesis 4).

For mediation test, we followed Baron and Kenny’s (1986) suggestion. First step, the direct effect from independent variable X (proactive personality) to the outcome Y (adjustment) must be significant (hypothesis 1a and 1b). Second step, the independent variable X (proactive personality) should be a significant predictor of the mediator M (psychological availability), which were also predicted by hypothesis 2a and 2b. Third step, to confirm the mediation effect, the effects of independent variable X (proactive personality) and mediator M (psychological availability) on outcome Y (adjustment) should be examined (hypothesis 3a and 3b). Moreover, we also ran Sobel’s test to check whether it is a full mediation or partial mediation.

For moderation test, we predicted that supportive supervisor relations would moderate the relationship between proactive personality and psychological availability in hypothesis 4. According to Aiken and West (1991) a moderated regression analysis is appropriate for testing the effect.

This study tested mediation effect and moderation effect by the PROCESS model in a bootstrap approach, developed by Hayes (2013), which is an add-on of SPSS.

### Ethics Statement

Following the 2013 revision of Helsinki Declaration, we designed our research to emulate a medical research study. The study was reviewed and approved by the Zhejiang University’s Global Entrepreneurship Research Centre ethics committees: Dr. Wang Wei, and Dr. Shao Yixuan. The data was volunteered by our studies participants and all research participants provided written and informed consent. They gave their responses after they were provide amble information on the studies parameters and we assured them that their responses were private and anonymous; they were under no pressure to respond to the researcher immediately. Additionally, every participant consent was obtained after they were provided information on the “aims, methods, duration of the questionnaires, sources of funding, any possible conflicts of interest, institutional affiliations of the researcher, the anticipated benefits and potential risks of the study and the discomfort it may entail”; between information and consent stage we gave every participant at least 48 h to think about whether to consent or not. Moreover, we confirmed that our research was conducted in an independent and unbiased manner.

## Results

**Tables 1** and **2** presents means, standard deviations, reliabilities, and correlations among the variables of SIE and HCN samples respectively.

**Table 1:** Means, standard deviations, reliabilities, and correlations of SIE samples.

Variables	*M*	*SD*	1	2	3	4	5	6	7	8	9
(1) Proactive relationship	5.48	0.74	(0.82)								
(2) Supportive supervisor relations	4.60	1.40	0.26^∗∗^	(0.93)							
(3) Psychological availability	5.59	0.85	0.48^∗∗^	0.33^∗∗^	(0.85)						
(4) Work adjustment	4.30	1.11	0.41^∗∗^	0.45^∗∗^	0.31^∗∗^	(0.88)					
(5) Interaction adjustment	4.19	1.08	0.42^∗∗^	0.45^∗∗^	0.30^∗∗^	0.64^∗∗^	(0.99)				
(6) Dyadic tenure	0.41	0.49	0.11	–0.10	0.05	–0.23^∗∗^	–0.17^∗^	–			
(7) Peer relationship	2.07	0.92	0.00	–0.11	–0.15	–0.14	–0.25^∗^	0.27^∗∗^	–		
(8) HCN as supervisor	0.83	0.36	–0.07	0.05	0.04	0.09	0.07	–0.27^∗∗^	–0.22^∗∗^	–	
(9) Length of SIEs	1.27	0.62	0.32^∗∗^	0.16	0.25^∗∗^	0.06	0.12	0.15	0.20^∗^	–0.09	–

*^∗^*p* < 0.05, ^∗∗^*p* < 0.01, in brackets show the reliability coefficient.*

**Table 2:** Means, standard deviations, reliabilities, and correlations of HCN samples.

Variables	*M*	*SD*	1	2	3	4	5	6	7	8
(1) Proactive relationship	6.50	0.64	(0.82)							
(2) Supportive supervisor relations	5.17	1.53	0.41^**^	(0.93)						
(3) Psychological availability	4.06	0.70	0.22^**^	0.27^**^	(0.85)					
(4) Work adjustment	3.97	1.01	0.42^**^	0.52^**^	0.45^**^	(0.88)				
(5) Interaction adjustment	3.57	1.20	0.33^**^	0.55^**^	0.31^**^	0.59^**^	(0.99)			
(6) Dyadic tenure	0.52	0.58	–0.02	0.09	–0.11	–0.14	–0.13	–		
(7) Peer relationship	2.24	0.99	0.12	–0.09	–0.10	–0.18^*^	–0.29^**^	0.14	–	
(8) HCN as supervisor	0.69	0.40	–0.10	–0.03	0.01	0.07	0.09	–0.12	–0.37^**^	–

*^∗^*p* < 0.05, ^∗∗^*p* < 0.01, in brackets show the reliability coefficient.*

### Test Mediation Effect

The Hypothesis 1a and 1b of this study were to examine the relationship between proactive personality and work and interaction adjustment. According to **Table 3**, SIEs’ proactive personality was positively and significantly related to work adjustment (*β* = 0.57, *t* = 5.77, *p* < 0.001, *CI* [0.378, 0.772]) and interaction adjustment (*β* = 0.61, *t* = 6.30, *p* < 0.001, *CI* [0.418, 0.801]). And, HCN’s proactive personality was positively related to work adjustment (*β* = 0.60, *t* = 4.76, *p* < 0.001, *CI* [0.352, 0.861]) but negatively related to interaction adjustment (*β* = -0.45, *t* = -2.55, *p* < 0.05, *CI* [-0.815, -0.099]). Therefore, Hypothesis 1a and 1b were supported.

**Table 3:** Regression results for mediation effect (SIE and HCN samples).

			Adjustment (SIEs/HCNs)							
Path estimated	Psychological availability		Work adjustment				Interaction adjustment			
Hypotheses	H2		H1		H3		H1		H3	
	Effect	*SE*	Effect	*SE*	Effect	*SE*	Effect	*SE*	Effect	*SE*
Dyadic tenure	–0.03	(0.09)	–0.08	(0.14)	–0.05	(0.12)	–0.16	(0.25)	0.19	(0.15)
Dyadic tenure^a^	0.44^**^	(0.13)	–0.22	(0.14)	–0.20	(0.16)	0.42^*^	(0.24)	0.47^*^	(0.25)
Peer relationship	0.21^*^	(0.10)	–0.08	(0.10)	–0.09	(0.11)	0.33^*^	(0.14)	–0.15	(0.08)
Peer relationship^a^	–0.18	(0.15)	0.18	(0.17)	0.07	(0.18)	0.29	(0.23)	0.23	(0.24)
HCN as supervisor	0.11	(0.18)	–0.03	(0.18)	0.02	(0.21)	–15	(0.08)	0.07	(0.15)
HCN as supervisor^a^	–0.21^*^	(0.10)	0.06	(0.15)	–0.11	(0.09)	–0.27^*^	(0.16)	–0.35^*^	(0.16)
Length of SIEs	–0.24^*^	(0.11)	0.30	(0.11)	0.42^**^	(0.12)	0.19	(0.15)	0.32^*^	(0.13)
Proactive personality	0.54^**^*	(0.08)	0.57^**^*	(0.09)	0.27^**^	(0.10)	0.61^**^*	(0.09)	0.23^**^	(0.08)
Proactive personality^a^	0.51^**^*	(0.11)	0.60^**^*	(0.12)	0.35^**^	(0.13)	–0.45^*^	(0.17)	–0.71^**^*	(0.19)
Psychological availability					0.54^**^*	(0.08)			0.67^**^*	(0.07)
Psychological availability^a^					0.48^**^*	(0.12)			0.49^**^	(0.19)
Overall*R*^2^	0.21		0.28		0.25		0.26		0.29	
Adjusted*R*^2^	0.14		0.18		0.15		0.16		0.19	

*^∗^*p* < 0.05, ^∗∗^*p* < 0.01, ^∗∗∗^*p* < 0.001, *^a^*Rated by HCNs.*

Hypothesis 2a and 2b investigated the relationship between proactive personality and psychological availability. The **Table 3** presents that, SIEs’ proactive personality was positively and significantly related to psychological availability (*β* = 0.54, *t* = 6.35, *p* < 0.001, *CI* [0.377, 0.719]). Therefore, Hypothesis 2a was supported. Also, **Table 3** displays that, HCN’s proactive personality was positively and significantly related to psychological availability (*β* = 0.51, *t* = 4.52, *p* < 0.001, *CI* [0.286, 0.738]). Therefore, hypothesis 2a and 2b were supported.

The Hypothesis 3a and 3b examined the relationship between proactive personality and work and interaction adjustment through psychological availability as a mediator. According to our results, the total effect of proactive personality on work adjustment from SIE’s perspective was significant (*β* = 0.57, *t* = 5.77, *CI* [0.378, 0.772]) also on interaction adjustment was positively significant (*β* = 0.61, *t* = 6.30, *p* < 0.001, *CI* [0.418, 0.801]). We found the indirect effect of proactive personality on work adjustment through psychological availability from SIE samples was (*Effect* = 0.29, *Boot SE* = 0.07, 95%*CI* [0.168, 0.475]), (Sobel *z =* 4.39, *p* < 0.00), indicating a full mediation; We also found indirect effect of proactive personality on interaction adjustment through psychological availability (*Effect =* 0.37, *Boot SE* = 0.08, 95%*CI* [0.217, 0.548]), (Sobel *z =* 5.12, *p* < 0.00), indicating a full mediation. Therefore, hypothesis 3a was supported. Also, the total effect of proactive personality on work adjustment from HCN’s perspectives was positively significant (*β* = 0.60, *t* = 4.76, *p* < 0.001, *CI* [0.352, 0.861]) but on interaction adjustment was negatively significant (*β* = -0.45, *t* = -2.55, *p* < 0.01, *CI* [-0.815, -0.099]). As well, we found the indirect effect of proactive personality on work and interaction adjustment through psychological availability HCN’s samples was (*Effect* = 0.24, *Boot SE* = 0.10, 95%*CI* [0.094, 0.525]), (Sobel *z =* 2.84, *p* < 0.00) and (*Effect* = 0.25, *Boot SE* = 0.13, 95%*CI* [0.032, 0.573]), (Sobel *z =* 2.20, *p* < 0.01), respectively. Both of them were fully mediated. Thus, Hypothesis 3b was supported.

### Test Moderation Effect

**FIGURE 2:**
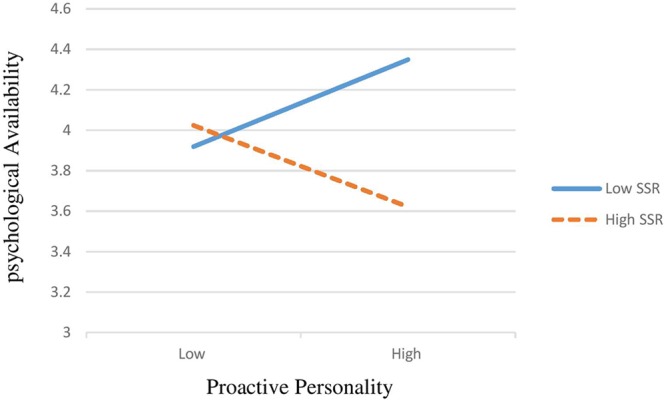
Interactive effects of the proactive personality and their supportive supervisor relations on psychological availability (SIE samples).

**FIGURE 3:**
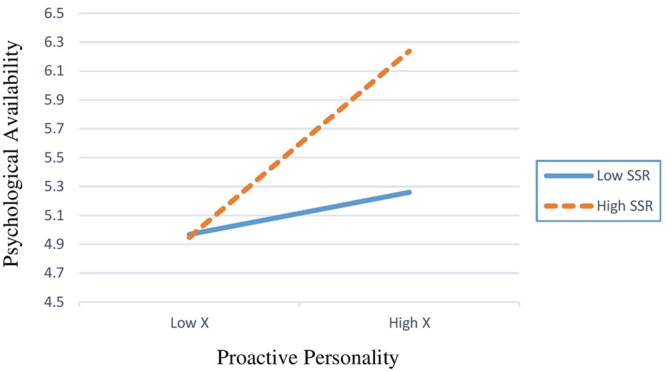
Interactive effects of the proactive personality and their supportive supervisor relations on psychological availability (HCN samples).

Hypothesis 4 predicted the relationship between proactive personality and psychological availability would be weakened or strengthened when SIEs and HCNs received low or high level of support from their supervisor. The **Table 4** shows that, from the SIE’s perspectives the coefficient of the interaction was 0.28 (95%*CI* [0.056, 0.518]), while from HCN’s perspectives it was 0.82 (95%*CI* [0.520, 1.235]). These results represented that supportive supervisor relations positively moderated the effect of proactive personality on psychological availability from both SIEs and HCNs perspectives. Thus, hypothesis 4 was supported. The conditional effect varied at different levels of supportive supervisor relations from SIEs perspectives (-1 SD as Low: 3.91; +1 SD as High: 4.34); HCN’s perspectives (-1 SD as Low: 4.96; +1 SD as High: 6.23). Also, **Figure 2** showed the interaction effects of the proactive personality and supportive supervisor relations on psychological availability from SIEs, and **Figure 3** showed the interaction effects of the proactive personality and supportive supervisor relations on psychological availability from HCN’s perspectives, both results displayed that the relationship between proactive personality and psychological availability would be strengthened when SIEs and HCNs received high support from their supervisor relations.

**Table 4:** Regression results for moderation effect (SIEs and HCNs sample).

Path estimated	Psychological availability	
Hypotheses	H4	
	Effect	*SE*
Dyadic tenure	0.29^*^	0.13
Dyadic tenure^a^	0.64^*^	0.15
Peer relationship	0.03	0.10
Peer relationship*^a^*	–0.07	0.17
HCN as manager	0.21	0.19
HCN as manager*^a^*	0.05	0.27
Length of SIEs	0.01	0.12
Proactive personality	0.00	0.09
Proactive personality*^a^*	0.53^**^*	0.10
Supportive supervisor relations	–0.11^*^	0.04
Supportive supervisor relations^a^	0.28^**^	0.09
ProacPer × SupSuperRela	–0.19^**^	0.06
ProacPer × SupSuperRela*^a^*	0.41^**^	0.14
*R*^2^	0.26	
*R*^2a^		0.34

*^∗^*p* < 0.05, ^∗∗^*p* < 0.01, ^∗∗∗^*p* < 0.001, *^a^*Rated by HCNs.*

## Discussion

The core contribution of this study was focusing on the research question of how SIEs and their local colleagues with high proactive personality can have a direct effect on their adjustment. Moreover, it discussed how the level of support received from a supervisor relationship, can act as a factor and can lead to sense of psychological availability between SIEs-HCNs, which in turn can enhance adjustment during their work and interactions.

### Theoretical Implications

The first theoretical contribution of this study is that we extended Kahn’s (1990) psychological availability concept by investigating the degree of availability between two actors, as a key issue of coping mechanisms which mediated the relationship between proactive personality and adjustment. Our hypothesis 3a’s and 3b’s results supported this mechanism and found a significant though indirect relationship through psychological availability between proactive personality and adjustment. Although cross-cultural research has previously linked proactive personality to adjustment (e.g., Ren et al., 2014), this study is the first to claim that psychological availability mediates this relationship to help to understand how to enhance the availability of SIEs and their local colleagues in turns to have a better adjustment. This can add additional support to SIE’s adjustment studies, during the challenges they face interacting with HCNs (e.g., Froese, 2012; Selmer and Lauring, 2014a). Therefore, the finding of the hypothesis 3a’s and 3b’s of this study support this proposition: if SIEs-HCNs behave proactively toward each other that can help to increase their levels of confidence and belief in their abilities (Hough and Schneider’s, 1996), in order to control the situation and overcome cultural barriers (Ashford and Black, 1996), this can help them to feel comfortable and have the mental capacity to be more available for each other, and in turn to have better adjustment.

Furthermore, in this study, our hypothesis 1b’s result displayed that if HCN behaves proactively without a sense of psychological availability with their SIEs colleague, they negatively effect their interaction adjustment with them. The previous literature which focused on the positive effect of proactive personality on individual’s adjustments (e.g., Kim et al., 2009; Li et al., 2014) ignored this factor and only view proactive personalities as a positive factor. For this reason, in this study, the concept of proactive personality with a focus on the relationship, when HCN proactively take action to build a relationship in the absence of investing available psychological resources with their SIEs colleague that may cause the SIEs to feel more stress and in turn to have negative interactions. Therefore, the results of this study represent the value of psychological availability as a mediator between proactive personality and adjustment, which is supported by the previous researchers (Finkel and Campbell, 2001; Danner-Vlaardingerbroek et al., 2013)

The second theoretical contribution is, this study extends the role of supervisor support (Liden et al., 1997; Kraimer et al., 2001) that can be leveraged to enhance psychological availability between foreigners and their local colleagues. Also, Kahn (1990) suggested that working in the insecure situation it’s a critical issue that may cause individuals to feel anxieties and leads them to lose their sense of availability for their partners. Moreover, this will hinder their abilities to maintain their focus on their roles. Therefore, we argued that supervisor support is facilitated that can help to provide a stable and secure environment (e.g., Cao et al., 2014; Nahum-Shani et al., 2014) and this can encourage both SIEs-HCNs to act proactively incapable to identifying and preventing potential problems that can help to control stress and lead to the sense of psychological availability. In support of this argument, our hypothesis 4’s result displayed that those SIEs-HCNs who received strong support from their supervisor they took more initiative in their relationship and were more eager to be available for each other. This outcome adds value of the role of supervisor support in SIE’s studies (Chen and Shaffer, 2017).

Finally, the global work situation has been recognized as a highly challenging situation full of new stressors. This is exacerbated by the fact that these actors share a cultural background (Lusha and Brian, 2001; Hsu, 2012). Indeed, in cross-cultural studies, we know less about how two actors at the same time adopt new coping strategies and interacting with each other. Most studies just spotlight SIEs (see Baruch and Forstenlechner, 2017 for a review). Although, in previous studies, it has been emphasized that both actors influence the interaction that is happening between them (e.g., Greenhaus et al., 2003; Hill, 2005; Danner-Vlaardingerbroek et al., 2013), impressively few studies have simultaneously observed both actors’ behavior in order to predict their mutual relationship aspects (e.g., Liu and Shaffer, 2005). But, this study fills this research gap by incorporating both SIEs- HCNs’ perspectives in the process of adjustment.

### Practical Implications

Working in the cross-cultural situation creates high amounts of stress, cultural confusion, strangeness, and emotional discomfort (Cao et al., 2013; Nolan and Morley, 2014) which may hamper SIEs-HCNs from being available to each other. Therefore, the hypothesis 4’s result displayed the quality of supervisor relations can play a critical role and encourage SIEs-HCNs to behave proactively, in order to enhance their perceived availability, to each other, and this may contribute to the body of international human resource management literature a study that will emphasize the importance of building good relationship between supervisors and the SIEs-HCNs dyads. Moreover, the lack of this support can undermine these relationships which create an insecure working environment and decrease the tendency to show initiatives between their SIEs-HCN. Conversely, it’s crucial for SIEs-HCNs to build a good relationship with their supervisors at work. This relationship can serve to relieve the stress and cultural confusion within the SIE-HCN dyad. This can increase their confidence and encourage them to behave proactively and lead to a sense of psychological availability among both SIE and HCN.

Adjustment between SIEs and their local colleagues has been considered as one of the important issues for global organizations, in order to build healthy and cooperative relationship among them as their competitive advantages (Froese and Peltokorpi, 2013; Nolan and Morley, 2014). Therefore, the hypothesis 3a’s and 3b’s results of this study can indicate that the quality and effectiveness of two-way of adjustment between SIEs-HCNs depends on their level of interest to being psychologically available with each other. Also, international human resource managers can utilize the model and results of this study as a coping mechanism between their SIEs-HCNs employees. They need to encourage or train their SIEs-HCNs employees how to act proactively or select ideal SIEs-HCNs who can behave proactively in their roles and relationship which this can help to enhance their interest in overcoming their cultural barriers and learn about each other, and they will feel comfortable being available to each other.

Finally, according to our control variable results, the length of SIE’s expatriation (in years) shows that those SIEs who stay longer in the host country or work in overseas are more capable of having better interactions and making better adjustments with their local colleagues. Therefore, the international human resource managers can select those SIEs who have long experience in overseas as the ideal candidate to work with their HCN employees. Also, the result of the dyadic tenure or the length of relationship in years from HCN’s perspective represented that those HCNs who have longer years working relationship with their SIEs partners are more eager to be psychologically available to them, as well as leading to better interactions with their SIEs.

### Limitations and Future Studies

There are several limitations of this study which we will be addressing in future studies. First, although, the research was investigating the adjusting strategy between SIEs and HCNs dyads, this may not be used to generalize to other types of dyads. Therefore, in future studies other may need to be extended to another kind of dyads, i.e., SIEs-supervisors. Secondly, in cross-cultural studies, there’s an emphasis on the fact that it takes time for individuals to adjust to the new cultural environment and local peoples (Furnham and Bochner, 1986; Black et al., 1991; Takeuchi, 2010; Firth et al., 2014). Also, Oberg (1960) suggested adaptation process with the new cultural situation by four stages or phases which can appear only at certain times, and during each phase, the individuals are experiencing difference difficulties and cultural shocks. Thus, this is very important issues for cross-cultural practitioners to identify how an individual adjustment occurs over time (Firth et al., 2014). But, this study was based on cross-sectional investigation and this may not give us a clear picture about the role of psychological availability between SIEs and HCNs during their adjustment in different phases or over the time. Therefore, future studies should pursue a longitudinal study in order to study the role of psychological availability on adjustment during different phases. Third, in this study, we focused on psychological availability in order to mitigate distinction between SIEs and their local colleagues and how this can help them to engage more fully and adjust to each other. But, we didn’t investigate how psychological availability cognitively, physically, emotionally effects this engagment, therefore, future research may need to take this matter under further consideration. Fourth, although, this study has controlled for length of relationship and peer relationship between SIEs and HCNs, in previous SIEs studies (Selmer and Lauring, 2010; Tharenou, 2010, 2013) they didn’t consider age and gender as key variables to be controlled for and in their studies, they found these variables have a high influence on SIEs adjustment, which in this study these control variables have been neglected. Also in our sample from our HCNs participant the majority of them are female and that may signal that HCN females are more able to psychologically engage with their foreign colleagues than males. In the future studies other may wish to consider this issue. Finally, the model of this research was only conducted in a Chinese cultural context and we believe the results of this model will be different if they are conducted in another cultural context, therefore, we suggest in future research investigating the same model in another country or within different cultural context.

## Conclusion

This paper reveals four main findings. First, the SIE’s/HCN’s proactive personality is linked to their better adjustment within that dyad. Secondly, the SIEs/HCNs with a high level of proactive personality are related to higher levels of psychological availability between each other. Third, for SIEs, psychological availability mediate the relationships between their proactive personality and adjustment with their HCN colleagues. Fourth, Relationship between proactive personality and psychological availability would be weakened/strengthened when SIEs and HCNs received low/high support from their supervisor. As a result, this study displayed the value of psychological availability as a coping mechanism between SIEs-HCNs which in turns leads them to better adjust to the new colleague. Besides, the direct effect of proactive personality as an antecedent and the indirect effect of supportive supervisor relations as a moderator, can leads SIEs/HCNs to have higher levels psychological availability between each other. Thus, the implication of this study is to help SIEs and HCNs in terms of perceiving different situations/environments and to provide guidance on how to cope with each other.

## Author Contributions

MJ focused on the theoretical foundation, model development, research design, data collection, and data analysis. ZW focused on the theoretical foundation. JM focused on data collection and editing the manuscript. BZ focused on data collection and data analysis.

## Conflict of Interest Statement

The authors declare that the research was conducted in the absence of any commercial or financial relationships that could be construed as a potential conflict of interest.
